# Outcomes of same-day discharge in bariatric surgery

**DOI:** 10.1007/s00464-024-11053-w

**Published:** 2024-07-19

**Authors:** Sydney Cooper, Shivam Patel, Matthew Wynn, David Provost, Monique Hassan

**Affiliations:** Department of Bariatric Surgery, Baylor Scott & White Hospital – Temple, Baylor College of Medicine, 2401 S 31St St MS-01-712, Temple, TX 76508 USA

**Keywords:** Same-day bariatric surgery, Robotic bariatric surgery, Bariatric surgery complications, Roux-en-Y gastric bypass, Sleeve gastrectomy

## Abstract

**Background:**

Restrictions during the COVID-19 pandemic influenced a shift to same-day discharge in bariatric surgery. Current studies show conflicting findings regarding morbidity and mortality. We aim to compare outcomes for same-day discharge versus admission after bariatric surgery.

**Methods:**

Subjects included patients who underwent primary laparoscopic or robotic-assisted sleeve gastrectomy or Roux-En-Y gastric bypass at an academic center. The inpatient group included patients discharged postoperative day one, and the outpatient group included patients discharged on the day of surgery. Primary outcomes included the number of emergency room visits, reoperations, IV fluid treatments, readmissions, and mortality within 30 days. Secondary outcomes were morbidity, including skin and soft tissue infection, pulmonary embolism, and acute kidney injury.

**Results:**

1225 patients met the inclusion criteria. In the gastric sleeve group, 852 subjects were outpatients and 227 inpatients. In the gastric bypass group, 70 subjects were outpatients, and 40 were inpatients. The mean age was 44.63 (17.38–85.31) years, and the mean preoperative BMI was 46.07 ± 8.14 kg/m^2^. The subjects in the outpatient group had lower BMI with fewer comorbidities. The groups differed significantly in age, BMI, and presence of several chronic comorbidities. The inpatient and outpatient groups for each surgery type did not differ significantly regarding reoperations, IV fluid treatments, or 30-day mortality. The inpatient sleeve group demonstrated a significantly higher readmission percentage than the outpatient group (4.6% vs 2.1%; p = 0.02882). The inpatient bypass group showed significantly greater ER visits (21.7% vs 10%; p = 0.0108). The incidence of adverse events regarding the secondary outcomes was not statistically different.

**Conclusion:**

Same-day discharge after bariatric surgery is a safe and reasonable option for patients with few comorbidities.

Bariatric surgery has been an effective treatment for obesity and obesity-related comorbidities [1, 2]. Though several procedures are offered, the most common operations performed today include laparoscopic sleeve gastrectomy and laparoscopic Roux-en-Y gastric bypass. The traditional length of stay for bariatric surgery before the COVID-19 pandemic involved at least one night in the hospital, potentially two to three at our institution. To save beds, staff, and resources, the COVID-19 pandemic restricted outpatient elective surgery that required inpatient admission in March 2020. These restrictions heavily impacted the ability to perform bariatric surgery, as hospitalization has historically been an overnight admission at a minimum, as was the standard at our institution. Therefore, these restrictions spurred a paradigm shift from admitting patients postoperatively to ambulatory bariatric surgery. Those who qualified for same-day discharge (SSD) would undergo a short post-operative observation period and then be discharged the same day if they met specified criteria. However, this trend continued even when surgeries requiring inpatient admission were no longer restricted.

The optimal length of stay after bariatric surgery has been studied extensively, focusing primarily on the safety of discharge on post-op day one compared to more extended hospital stays [3, 4]. Since the onset of COVID-19, SDD for bariatric surgery has become more prevalent and has shown to be feasible [5].

Postoperative risks of bariatric surgery include staple line leak (primarily in sleeve gastrectomy), bowel obstruction, deep vein thrombosis, GI bleeding, infection, and hernia. The mortality rate for bariatric surgery is estimated to be 0.03–0.2%, and the 30-day risk of adverse events is less than 6% on average [2]. In general, gastric bypass is associated with more adverse events, hospitalizations, and interventions, which may explain the increased risk of mortality [2]. Despite these risks, bariatric surgery is increasingly being performed outpatient, and more studies regarding outcomes are being conducted.

Our study aims to investigate whether patient outcomes are similar for same-day discharge versus admission for laparoscopic or robotic-assisted bariatric procedures at our tertiary care facility among select patients with a specific post-operative recovery protocol.

## Methods

### Study design

The study is a retrospective cohort analysis of patients discharged on the same day as surgery versus patients discharged on POD 1 from July 2019 to March 9th, 2023. The POD 1 group included patients who underwent surgery from July 2019 to June 2020 and were discharged postoperative day 1 (POD1). Since SDD became more prevalent after the onset of COVID-19, the SDD group included patients who underwent surgery between July 2020 and March 2023. Patients discharged after POD1 or underwent revisional surgeries were excluded from the study. Patient data was obtained through a chart review, and the Institutional Review Board approved the study.

### Study aims

The study's primary outcomes included readmission rates, emergency room visits, outpatient intravenous fluid treatment within 30 days of surgery, and 30-day mortality rates. Secondary outcomes included immediate postoperative complications within 30 days, including leaks, surgical site infections, pneumonia, urinary tract infections (UTI), the need for blood transfusion, or the development of venous thromboembolism (VTE).

### Criteria for same-day discharge

Given the multiple comorbidities associated with obesity, patients only qualified for same-day discharge if certain criteria developed at our specific institution were met in the pre and perioperative period. Preoperatively, patients with BMI > 60, home oxygen use, chronic kidney disease stage five, and high VTE risk were not eligible. Patients also were only permitted to be discharged if certain milestones were met in the recovery area. The protocol and milestones were identical for both gastric sleeve and gastric bypass. The milestones included pain well-controlled on oral medications, remaining alert, interactive, and upright in a chair, ambulating three times, performing incentive spirometry, tolerating 30 ml of water every fifteen minutes, maintaining a stable heart rate under 100 beats per minute, and ability to void. The milestones were tracked as a checklist on a board in the patient’s recovery room. Failure to meet these discharge criteria by the time the recovery unit closed at approximately 10 pm necessitated admission overnight. Those patients who were admitted after failing to meet the milestones were included in the POD1 group in the data analysis.

### Same-day discharge pathway

All patients undergoing same-day discharge were placed on a specific recovery pathway designed by our bariatric surgeons and inpatient coordinators. In order to provide access to this critical surgery for patients, the recovery pathway was developed in response to the COVID pandemic limiting inpatient beds for elective surgeries. The pathway began at the initial surgical consultation to determine eligibility for same-day discharge. Patients deemed eligible received additional education focused on procedure-specific expectations, post-operative care, oral prescription management, and pain control plans. On the day of surgery, patients received aprepitant, gabapentin, acetaminophen, celecoxib, and venous chemoprophylaxis preoperatively. Intraoperatively, patients received at least one liter of crystalloid and either direct port site infiltration of local anesthetic or via transversus abdominis plane (TAP) block based on the surgeon’s preference. In recovery, patients received intravenous fluids at 200 ml per hour and continued multimodal pain control consisting of acetaminophen, celecoxib, and as-needed tramadol.

### Data collection

Data was collected using the bariatric department MBSAQIP database, which contains data points compiled from individual electronic medical record chart reviews.

### Statistical analysis

Continuous variables were reported using the mean and standard deviation, while categorical variables used frequencies and percentages. A comparison of continuous variables was made using the Wilcoxon test, while a comparison of categorical variables was made using the Chi-Squared test. All analyses were performed using SAS 9.4. The significance level was set at 0.05.

## Results

### Patient demographics

A total of 1224 patients met the inclusion criteria. There were 940 (76.7%) patients in the same-day discharge group. In the sleeve gastrectomy group, there were 1108 patients. Eight hundred seventy subjects were outpatients, and 238 were inpatients. There were 116 patients in the Roux en Y gastric bypass group, consisting of 70 outpatients and 46 inpatients. Six hundred twenty-four patients (50.9%) underwent surgery laparoscopically, and 600 patients (49.1%) underwent surgery robotically.

There were 1,027 (83.9%) females in the study and 197 (16.1%) males, with a mean age of 44.6 years. The mean BMI close to surgery was 46.1 kg/m^2^. Associated comorbidities included diabetes (n = 314, 25.6%), dyslipidemia (n = 513, 41.9%), COPD (n = 13, 1.1%), preoperative sleep apnea (n = 698, 57%), and hypertension (n = 662, 54.1%). Forty-eight patients were active smokers. Table 1 summarizes the patient demographics between same-day discharge and hospitalized patients for laparoscopic gastric sleeve and laparoscopic Roux en Y gastric bypass surgeries.

### Operative and outcome results

There was a statistically significant difference in the mean operative time between the same-day discharge group and the hospitalized group for Roux en Y gastric bypass only (*p* = 0.0194); there was no difference between the operative times for the sleeve gastrectomy group.

The primary outcomes assessed in the study were the thirty-day readmission rate, emergency room visits, outpatient intravenous fluid treatment, and thirty-day mortality. For readmissions, there was a greater proportion of POD1 patients in the sleeve gastrectomy group (2.7% SDD, 4.6% POD1, p = 0.02882), which was statistically significant (Table 2). For the bypass group, the readmission rate was 0% for SDD and 13.1%, although this was not statistically significant due to insufficient sample size. For ER visits, there was a greater proportion of POD1 patients in the sleeve gastrectomy group (9.2% SDD, 11.3% POD1), though this was not statistically significant (p = 0.3199). In the bypass group, the ER visits were significantly greater in the POD 1 group (8.6% SDD, 26% POD1; p = 0.0108). There was no statistically significant difference between groups for patients receiving intravenous treatment as an outpatient.

For secondary outcomes, the sleeve gastrectomy SDD group had a higher incidence of adverse events than the POD 1 group. However, there were no statistically significant differences between the SDD and POD1 groups for both sleeve gastrectomy and Roux-en-Y gastric bypass groups for the secondary outcomes. Table 3 summarizes the primary and secondary outcomes of the study.

## Discussion

Outpatient bariatric surgery could be the new standard as we advance. In that case, it is now essential to quantify whether outcomes are at least equivalent when patients undergo bariatric surgery as an outpatient with a short post-operative observation period in the recovery room. Outcomes will be estimated based on mortality, emergency room visits, readmissions, reoperations, and surgical complications. If outcomes are not inferior, this may change the standard post-operative care for bariatric surgery.

In the past several years, studies assessing outcomes for same-day discharge after bariatric surgery have increased [6, 7], with conflicting conclusions regarding morbidity and mortality for SDD [8–10]. A retrospective study identified outcomes for patients who underwent SDD bypass after meeting a specific inclusion criterion and noted no significant complications and no deaths [11]. Elnahas et al. performed a retrospective study on gastric bypass surgery and compared outcomes from POD 1 versus POD 2; there was no statistically significant difference in adverse events, reoperation rates, or overall 30-day complication rate [3]. Conversely, Morton et al. reported that patients with SDD and discharge on POD1 were associated with an increased risk of 30-day mortality [4]. Similarly, Inaba et al. also reported an increase in 30-day mortality in the SDD group for RYGB, along with higher rates of morbidity, cardiac arrest requiring resuscitation, unplanned intubation, unplanned admission to the intensive care unit, and renal injury [9].

For sleeve gastrectomy, mortality, readmission rates, ED visits, staple line leaks, and SSI between the SDD and POD1 groups are similar without statistically significant findings in a study by Al-Masrouri [5]. In a randomized controlled trial, patients eligible for SDD were compared with those discharged on POD 1, and outcomes also showed no significant differences between the two groups [12]. In contrast, Khorgami et al. and Inaba et al. showed increased readmission rates, reoperation rates, infection rates, and bleeding rates amongst the SDD groups without any increased mortality [8, 10].

A meta-analysis that consisted of 14 retrospective studies with 33,403 patients who underwent SDD sleeve gastrectomy and RYGB reported a high success rate for SDD (sleeve gastrectomy: 99% and RYGB: 88–98%) with low complication and mortality rates. Most studies had similar age, BMI, and ASA classification criteria, owing to the importance of patient selection in SDD surgeries [6]. Patient selection is the most critical factor in predicting outcomes, although no standardized criteria exist.

At our institution, the focus has been developing an algorithm that identifies patients who meet the criteria for discharge on the day of surgery based on the presence or absence of certain risk factors. Some studies have shown that predictors for longer hospitalization in patients undergoing surgery include patient factors such as diabetes, COPD, renal insufficiency, and operative time; BMI and age did not predict longer hospitalization [13]. In patients who underwent SDD for surgery, associated factors that predicted the risk for readmission include female sex, gastric reflux, renal insufficiency, and intraoperative drain placement [14]. Patients must have a BMI of 40 kg/m^2^ or 35 kg/m^2^ with comorbidities associated with obesity to be a candidate for bariatric surgery [2]. As a result, bariatric surgery patients are more likely to have diabetes, dyslipidemia, hypertension, or sleep apnea, which may complicate postoperative care.

Our study is a retrospective cohort analysis where patients were either admitted to the hospital or discharged on the same day as surgery. Patients were eligible for SDD unless they were of BMI > 60, required home O2 use, had chronic kidney disease stage five, and had a high risk for venous thromboembolism. A post-operative protocol was developed in order to ensure safe discharge for those who were eligible for SDD. SDD would only occur if patients met specific postoperative milestones in the recovery unit. These milestones included pain well-controlled on oral medications, ambulating three times around the unit, using incentive spirometry, tolerating 30 ml of water every fifteen minutes without nausea or vomiting, remaining normocardic under 100 beats per minute, and voiding independently. Additionally, patients were assessed by a physician and periodically by nursing to ensure they were sitting independently in a chair, remaining alert, appropriately interacting with nursing and providers, and showing no signs of somnolence or altered mental status. Failure to meet these specific milestones would result in admission overnight and discharge the following day.

Our single-center study showed only statistically significant findings in readmission rates in the inpatient sleeve gastrectomy group and the number of emergency room visits in the inpatient gastric bypass group. This is encouraging regarding the safety of discharge on the day of surgery.

In our study, there were significant differences between the SDD group for sleeve gastrectomy, with POD1 patients being older, having a higher BMI, and having obesity-related comorbidities like diabetes, sleep apnea, hypertension, hyperlipidemia, and a history of myocardial infarction. Patients in the bypass group significantly differed in age and history of deep vein thrombosis; otherwise, there were no significant differences, likely due to the lower sample size compared to the sleeve gastrectomy group.

The most significant limitation of the study is that it was performed at a single center among only two surgeons. Therefore, future studies are needed to compare outcomes amongst surgeons at multiple centers to assess the impact of specific techniques and protocols. Additionally, though there were 1224 subjects enrolled, the sample size still needed to be increased in some cases to draw statistical conclusions. For instance, the readmission rate for the robotic gastric bypass subgroup that was same-day discharge was five patients compared to only 1 or 0 for the other three gastric bypass subgroups, but still not statistically significant. This was a limitation when investigating several secondary outcomes as well. Finally, selection and recall bias are limitations due to the study's retrospective nature. Patients were not randomized into each arm, and as seen in Table 1, patients undergoing sleeve gastrectomy with a higher BMI, male sex, and older age were more likely to be kept overnight in the POD1 discharge group. In addition, patients who did not meet discharge criteria and were kept overnight were automatically included in the POD1 group, as this data was collected retrospectively. Further studies investigating why patients crossed over from the SDD group to the POD1 group and specifically which milestones were most often not met could be helpful for developing an evidence-based protocol to potentially decrease the number of patients crossing over, subsequently increasing the safety of SDD. Robotic-assisted surgeries have been reported to have an increased mean operative time and increased hospital stays despite similar outcomes compared to the laparoscopic group. However, many of the studies have limitations in terms of quality [15–18]. Kannan et al. reported in their research that there was no significant difference in operative time between laparoscopic and robotic-assisted sleeve gastrectomy groups and a shorter length of stay in the robotic group (*p* = 0.01) [19]. Of note, none of the studies analyze the outcomes of ambulatory robotic-assisted surgeries. Although our study was not adequately powered to demonstrate a difference in the SDD and POD1 groups, this may be a future direction that would focus on outcome differences between laparoscopic versus robotic-assisted techniques.

## Conclusion

Same-day discharge is a safe option for patients undergoing bariatric surgery. However, patients must be carefully selected, and stringent discharge criteria must be utilized. If standardized criteria are implemented, positive outcomes can be achieved widely for ambulatory bariatric surgery.

## Appendix

See Tables

**Table 1:** Patient characteristics and demographics

	SG^a^			RNYGB^b^		
	SDD^c^	POD1^d^	*P*	SDD	POD1	*P*
Age (yr), mean ± SD	42.6 ± 11.5	50 ± 14.3	< 0.0001	47.2 ± 9.9	51.9 ± 10.4	0.0325
Male sex, no. (%)	124 (14.3%)	57 (23.9%)	0.003	11 (15.7%)	5 (10.9%)	0.4592
Height (cm), mean	165.3	166.3	0.1227	165.3	165.4	0.3895
Highest weight (kg), mean	128.8	141.4	< 0.0001	127.6	123.8	0.1241
Highest BMI^e^, mean	46.6	50.8	0.0002	45.8	45	0.3959
Preop weight (kg), mean	124.9	135.8	< 0.0001	124.2	121.6	0.1686
Preop BMI, mean	45.5	49	0.0008	44.6	44.2	0.5490
Preop Cr^f^, mean	0.8	1.2	< 0.0001	0.9	0.9	0.0565
HbA1c, mean	6.3	6.9	0.0014	6.6	6.5	0.9562
Race, no. (%)						
Black/African American	222 (25.5%)	60 (25.2%)	–	6 (8.6%)	4 (8.7%)	–
White	580 (66.7%)	163 (68.5%)	–	60 (85.7%)	41 (89.1%)	–
Smoker, no. (%)	40 (4.6%)	7 (2.9%)	0.2612	1 (1.4%)	0	1.000
Diabetes, no. (%)	58 (6.7%)	33 (13.9%)	0.0004	5 (7.1%)	2 (4.3%)	0.7116
COPD^g^, no. (%)	5 (0.6%)	6 (2.5%)	0.0161	1 (1.4%)	1 (2.2%)	1.000
OSA^h^, no. (%)	457 (52.5%)	167 (70.2%)	< 0.0001	43 (61.4%)	31 (67.4%)	0.5133
History of PE^i^, no. (%)	4 (0.5%)	5 (2.21%)	0.0124	0	1 (2.2%)	0.2154
Preop DVT^j^, no. (%)	26 (3.0%)	24 (10.1%)	< 0.0001	2 (2.9%)	6 (13.0%)	0.0342
History of MI^k^, no. (%)	10 (1.1%)	12 (5%)	0.0001	0	1 (2.2%)	0.2154
Preop HTN^l^, no. (%)	430 (49.4%)	170 (71.4%)	< 0.0001	34 (48.6%)	28 (69.9%)	0.1939
Preop HLD^m^, no. (%)	338 (38.9%)	120 (50.4%)	0.0013	36 (51.4%)	19 (41.3%)	0.2854
Dialysis, no. (%)	0	8 (3.4%)	< 0.0001	0	0	-

^a^Sleeve gastrectomy

^b^Roux en Y gastric bypass

^c^Same day discharge

^d^Postoperative day 1

^e^Body mass index

^f^Creatinine

^g^Chronic obstructive lung disease

^h^Obstructive sleep apnea

^i^Pulmonary embolism

^j^Deep vein thrombosis

^k^Myocardial infarction

^l^Hypertension

^m^Hyperlipidemia

1,

**Table 2:** 30-Day readmission rate for sleeve gastrectomy

	No Readmission (row %) (column %)	Readmission of at least 1 day (row %) (column %)	Row total	*P*
SDD^a^	852 (97.9%), (79%)	18 (2.1%), (62.1%)	870	.02882
POD1^b^	227 (95.4%), (21%)	11, (4.6%), (37.9%)	238	
Total	1079	29	1108	

^a^SDD

^b^POD1

^2^,

**Table 3:** Summary of primary and secondary outcomes for both SDD and POD1 patients in both surgical groups

	SG^a^			RYGB^b^		
	SDD^**c**^	POD1^**d**^	*P*	SDD	POD1	*P*
Readmission, no. (%)	18 (2.1%)	11 (4.6%)		0	6 (13.1%)	
ICU^e^ Admission, no. (%)	7 (0.8%)	3 (1.3%)		0	0	
ED^f^, no. (%) Visit	80 (9.2%)	27 (11.3%)	0.3199	6 (8.6%)	12 (26%)	0.0108
IV treatment as an outpatient, no. (%)	88 (10.1%)	17 (7.1%)	0.1654	9 (12.9%)	8 (17.4%)	0.4994
Reoperation, no. (%)	2 (0.2%)	1 (0.4%)		0	0	
PE^g^, no. (%)	3 (0.3%)	1 (0.4%)		0	0	
Post Op DVT^h^, no. (%)	3 (0.3%)	2 (0.8%)		0	0	
Superficial SSI^i^, no. (%)	2 (0.2%)	1 (0.4%)		0	1 (2.2%)	
Deep SSI^j^, no. (%)	2 (0.2%)	1 (0.4%)		0	1 (2.2%)	
Anastomotic Leak, no. (%)	1 (0.1%)	0		0	0	
AKI^k^, no. (%)	3 (0.3%)	0		0	0	
UTI^l^, no. (%)	12 (1.4%)	3 (1.3%)		1 (1.4%)	1 (2.2%)	
Cardiac Arrest requiring chest compressions, no. (%)	1 (0.1%)	0		0	0	
Newly diagnosed COVID-19, no. (%)	13 (1.5%)	2 (0.8%)		1 (1.4%)	0	

^a^Sleeve gastrectomy

^b^Roux en Y gastric bypass

^c^Same day discharge

^d^Postoperative day 1

^e^Intensive care unit

^f^Emergency department

^g^Pulmonary embolism

^h^Deep vein thrombosis

^i^Skin and soft tissue infection

^j^Acute kidney injury

^k^Urinary tract infection

3 and

**Table 4:** Primary and secondary outcomes divided by approach

	SG				RYGB			
	LSG^a^ SDD^c^	LSG POD1^d^	Robotic SG SDD	Robotic SG POD1	LRYGB^b^ SDD	LRYGB POD1	Robotic RYGB SDD	Robotic SDD POD1
Readmission, no. (%)	8 (1.82%)	6 (4.20%)	10 (2.32%)	5 (5.26%)	0	1 (7.69%)	0	5 (15.15%)
ICU^e^ Admission, no. (%)	6 (1.36%)	1 (0.7%)	1 (0.23%)	2 (2.11%)	0	0	0	0
ED^f^, no. (%) Visit	40 (9.09%)	16 (11.9%)	40 (9.3%)	11 (11.58%)	0	2 (15.38%)	6 (9.09%)	10 (30.3%)
IV treatment as an outpatient, no. (%)	41(9.32%)	6 (4.20%)	47 (10.93%)	11 (11.58%)	0	3 (23.08%)	9 (13.64%)	5 (15.15%)
Reoperation, no. (%)	0	0	0	0	0	0	0	0
PE^g^, no. (%)	0	0	3 (0.7%)	1 (1.05%)	0	0	0	0
Post Op DVT^h^, no. (%)	1 (0.23%)	2 (1.40%)	2 (0.47%)	0	0	0	0	0
Superficial SSI^i^, no. (%)	1 (0.23%)	1 (0.7%)	1 (0.23%)	0	0	0	0	1 (3.03%)
Deep SSI, no. (%)	1 (0.23%)	1 (0.7%)	1 (0.23%)	0	0	0	0	1 (3.03%)
Anastomotic Leak, no. (%)	0	0	1 (0.23%)	0	0	0	0	0
AKI^j^, no. (%)	1 (0.23%)	0	2 (0.47%)	0	0	0	0	0
UTI^k^, no. (%)	7 (1.59%)	1 (0.7%)	5 (1.16%)	2 (2.11%)	0	1 (7.69%)	1 (1.52%)	0
Cardiac Arrest requiring chest compressions, no. (%)	0	0	1 (0.23%)	0	0	0	0	0
Newly diagnosed COVID-19, no. (%)	7 (1.59%)	1 (0.7%)	6 (1.40%)	1 (1.05%)	0	0	1 (1.52%)	0

^a^Laparoscopic sleeve gastrectomy

^b^Laparoscopic Roux en Y gastric bypass

^c^Same day discharge

^d^Postoperative day 1

^e^Intensive care unit

^f^Emergency department

^g^Pulmonary embolism

^h^Deep vein thrombosis

^i^Skin and soft tissue infection

^j^Acute kidney injury

^k^Urinary tract infection

4.
